# Real-World Treatment Patterns and Safety Outcomes of Targeted Therapies in a Single-Center Chronic Lymphocytic Leukemia Cohort

**DOI:** 10.3390/medicina62040736

**Published:** 2026-04-12

**Authors:** Seda Jeral Evinç, Çağla Eyüpler Akmercan, Tarık Ercan, Fatma Arıkan, Meral Ulukoylu Mengüç, Tayfur Toptaş, Işık Atagündüz, Tülin Tuğlular, Asu Fergün Yılmaz

**Affiliations:** 1 Department of Medical Oncology, Cerrahpaşa Faculty of Medicine, Istanbul University-Cerrahpaşa, 34098 İstanbul, Türkiye; 2 Department of Medical Oncology, Istanbul Göztepe Prof. Dr. Süleyman Yalçın City Hospital, Istanbul Medeniyet University, 34722 İstanbul, Türkiye; cagla.eyupler@gmail.com; 3 Department of Hematology, Medical Park Göztepe Hospital, Bahçeşehir University, 34732 İstanbul, Türkiye; tarikercan86@gmail.com; 4 Department of Hematology, Faculty of Medicine, Marmara University, 34854 İstanbul, Türkiye; fatmagecgelarikan@gmail.com (F.A.); umeral@hotmail.com (M.U.M.); toptast@gmail.com (T.T.); isikkaygusuz@yahoo.com (I.A.); fergunaydin@hotmail.com (A.F.Y.); 5 Department of Hematology, Academic Hospital, 34718 İstanbul, Türkiye; ttuglular@gmail.com

**Keywords:** chronic lymphocytic leukemia, targeted therapy, real-world data, treatment patterns, drug safety

## Abstract

*Backgroundand Objectives:* Targeted therapies are increasingly used in the management of chronic lymphocytic leukemia (CLL); however, real-world data from routine clinical practice remain limited. *Materials and Methods:* We performed a retrospective analysis of 47 patients with CLL who received at least one targeted therapy at a tertiary university hospital. Clinical characteristics, treatment responses, and adverse events were assessed. A total of 58 treatment events were included. *Results:* Obinutuzumab, ibrutinib, and venetoclax were administered in 34.5%, 46.5%, and 19.0% of treatment events, respectively. Numerically higher response rates were observed in treatment events involving obinutuzumab compared with ibrutinib and venetoclax (92.9% vs. 54.5% and 55.6%, respectively); however, treatment allocation was not randomized and these findings should be interpreted descriptively. Median overall survival from initiation of the first targeted therapy was 30.9 months. Adverse events occurred in more than 80% of treatment events. Neutropenia was more frequent with obinutuzumab and venetoclax, whereas bleeding events were more common with ibrutinib. *Conclusions:* In this real-world cohort, targeted therapies showed response patterns and safety findings consistent with routine clinical practice. Obinutuzumab was more frequently prescribed in older and more comorbid patients, reflecting treatment patterns rather than comparative superiority. These findings should be considered descriptive and hypothesis-generating, given the retrospective and single-center design.

## 1. Introduction

Chronic lymphocytic leukemia (CLL) is the most common leukemia in adults and displays a remarkably heterogeneous clinical course [1]. Some patients remain asymptomatic for many years and never require treatment, whereas others develop progressive disease accompanied by cytopenias, organomegaly, or disease-related symptoms that necessitate systemic therapy [1]. Clinical decision-making in CLL is therefore rarely straightforward. Age, performance status, comorbidities, and underlying disease biology all contribute to treatment selection, making both the timing of therapy and the choice of treatment highly individualized [2].

In recent years, this individualized approach to CLL management has been further refined by the introduction of targeted therapies that interfere with key signaling pathways involved in leukemic cell survival. These agents have substantially altered treatment algorithms, particularly for patients who are older, frail, or burdened by comorbid conditions. Bruton tyrosine kinase inhibitors, BCL-2 inhibitors, and monoclonal antibodies targeting CD20 have demonstrated clinically meaningful improvements in disease control and survival compared with traditional chemoimmunotherapy, while offering a more favorable toxicity profile [3,4]. As a result, chemotherapy-free regimens have increasingly become a central component of treatment strategies across a broad spectrum of patients, reshaping real-world clinical practice and challenging historical treatment paradigms in CLL.

Obinutuzumab, a type II anti-CD20 monoclonal antibody, exerts its antileukemic activity through enhanced antibody-dependent cellular cytotoxicity, complement activation, and direct induction of apoptosis. Its clinical relevance was established in the CLL11 trial, where the combination of obinutuzumab and chlorambucil achieved superior progression-free survival and higher complete response rates compared with rituximab-based therapy in patients with significant comorbidities, leading to its incorporation into frontline treatment strategies for selected patients [5]. Venetoclax represents a mechanistically distinct approach by selectively inhibiting the antiapoptotic protein BCL-2, thereby restoring programmed cell death in malignant B cells. Initial studies demonstrated substantial activity in relapsed or refractory CLL, including patients with high-risk cytogenetic abnormalities such as del (17p) [6]. Long-term follow-up of the MURANO trial subsequently confirmed the durability of responses with fixed-duration venetoclax-based regimens in the relapsed/refractory setting, supporting its role as a key component of contemporary CLL management [7]. In the frontline setting, the CLL14 trial demonstrated that fixed-duration venetoclax-obinutuzumab improved outcomes in previously untreated patients with CLL and coexisting conditions, supporting its use as a first-line option in older and unfit patients [8]. Ibrutinib, the first-in-class Bruton tyrosine kinase inhibitor, disrupts B-cell receptor signaling and has shown consistent efficacy across multiple disease settings. Randomized clinical trials, including RESONATE in relapsed/refractory disease and RESONATE-2 in previously untreated older patients, established ibrutinib as an effective therapeutic option across different clinical settings. Nevertheless, its distinct toxicity profile underscores the importance of individualized treatment selection, particularly in routine clinical practice [9,10].

Despite the robust evidence generated by randomized clinical trials, data reflecting the use of targeted therapies in routine clinical practice remain limited. In particular, real-world outcomes may differ from trial results due to patient heterogeneity, comorbidity burden, and variations in treatment access. In this context, we conducted a retrospective analysis of patients with chronic lymphocytic leukemia treated with obinutuzumab, ibrutinib, and venetoclax at a single tertiary university hospital, aiming to evaluate treatment patterns and clinical outcomes, with a focus on safety in real-world care.

## 2. Materials and Methods

### 2.1. Patients

In this retrospective study, medical records of 596 patients diagnosed with chronic lymphocytic leukemia (CLL) at the Hematology Department of a tertiary university hospital were reviewed. All diagnoses were re-evaluated according to the 2018 International Workshop on Chronic Lymphocytic Leukemia (IWCLL) diagnostic criteria [2]. Patients who received at least one targeted therapy (obinutuzumab, ibrutinib, or venetoclax) and were followed for a minimum of three months were eligible for inclusion. A total of 549 patients were excluded due to the absence of targeted therapy exposure, incomplete medical records, or loss to follow-up. Ultimately, 47 patients who met all inclusion criteria were included in the final analysis. Demographic characteristics (age, sex, age at diagnosis), laboratory parameters (complete blood count, lactate dehydrogenase, β2-microglobulin), genetic analyses, comorbidity burden assessed by the Cumulative Illness Rating Scale (CIRS) and Charlson Comorbidity Index (CCI), physical examination findings, treatment characteristics, and treatment responses were retrospectively extracted from institutional medical records. Renal function was assessed using both the estimated glomerular filtration rate (eGFR), calculated by the Modification of Diet in Renal Disease (MDRD) equation, and creatinine clearance, estimated using the Cockcroft–Gault formula.

### 2.2. Treatment

In all patients, indications for treatment initiation were re-evaluated according to the 2018 International Workshop on Chronic Lymphocytic Leukemia (IWCLL) recommendations [2]. During the study period, 58 targeted therapy courses were administered, each analyzed as an independent treatment event for treatment-based analyses. Treatment events were classified as frontline when the targeted agent was used as first-line therapy for CLL and as relapsed/refractory when administered after at least one prior line of treatment.

#### 2.2.1. Obinutuzumab Treatment

During the study period, obinutuzumab–venetoclax was not routinely used in our center. Obinutuzumab was therefore administered either as monotherapy or in combination with chlorambucil (0.5 mg/kg on days 1–15), according to patient fitness, comorbidity burden, and physician judgment in routine clinical practice. Obinutuzumab was administered intravenously at a fixed dose of 1000 mg on days 1, 8, and 15 of the first cycle. In subsequent cycles, a dose of 1000 mg was administered on day 1 of each cycle. The first dose of the initial cycle was split into two infusions of 100 mg and 900 mg on days 1 and 2, respectively. All patients received standard premedication with dexamethasone (20 mg), paracetamol (1 g), and pheniramine. Tumor lysis syndrome prophylaxis with allopurinol (300 mg/day) was administered to all patients. The median number of administered treatment cycles was 6 (range, 1–8). Hepatitis B prophylaxis was provided to 25% of patients due to a prior history of infection. Primary prophylaxis with granulocyte colony-stimulating factor (G-CSF) was not routinely used; however, secondary G-CSF prophylaxis was administered in 40% of patients during treatment.

#### 2.2.2. Ibrutinib Treatment

Ibrutinib was administered orally at a dose of 420 mg/day and continued until disease progression or the development of unacceptable toxicity. Routine primary prophylaxis against viral, bacterial, or fungal infections was not administered. Two patients received secondary antiviral prophylaxis with valacyclovir due to recurrent herpes zoster infections. Hepatitis B prophylaxis was provided to seven patients (26%) based on a prior history of infection. Primary granulocyte colony-stimulating factor (G-CSF) prophylaxis was not routinely used. G-CSF was administered in two patients (7.5%) who developed neutropenia during treatment, with dose modifications applied as clinically indicated.

#### 2.2.3. Venetoclax Treatment

Venetoclax was initiated and administered according to the dose-escalation schedule recommended in the product information. Patients received venetoclax either as monotherapy or in combination with rituximab, which was administered intravenously at a dose of 375 mg on the first day of treatment, depending on treatment line and physician preference in routine clinical practice. In our cohort, venetoclax-based treatment was used predominantly in the relapsed/refractory setting. Primary hepatitis B prophylaxis was provided to 45.4% of patients based on a prior history of infection. No routine primary antiviral or antifungal prophylaxis was administered in patients treated with venetoclax. Primary granulocyte colony-stimulating factor (G-CSF) prophylaxis was not used; however, G-CSF was administered in 63.6% of patients who developed neutropenia during treatment, with appropriate dose modifications.

### 2.3. Treatment Safety and Adverse Events

Adverse events were graded and recorded according to the National Cancer Institute Common Terminology Criteria for Adverse Events (CTCAE), version 4.03 [11].

### 2.4. Treatment Response Evaluation

Treatment response was assessed according to the 2018 International Workshop on Chronic Lymphocytic Leukemia (IWCLL) criteria and categorized as complete response (CR), partial response (PR), stable disease (SD), or progressive disease (PD). Because this was a retrospective study and response assessment time points were not fully uniform in routine clinical practice, the best documented response achieved during treatment or before treatment discontinuation was recorded for each treatment event. Overall response rate (ORR) was defined as the proportion of treatment events achieving either complete or partial response. Response analyses were conducted on a per-treatment-event basis, allowing patients who received sequential targeted therapies to contribute more than one event to the analysis [2].

### 2.5. Survival Analysis Definitions

Overall Survival (OS) was defined as the time from the initiation of the first targeted therapy to death from any cause. Patients who were alive at the time of the last follow-up were censored on that date.

### 2.6. Ethical Approval

This study was reviewed and approved by the Local Clinical Research Ethics Committee on 5 November 2021 (Protocol No. 09.2021.1226).

### 2.7. Statistical Analysis

Statistical analyses were performed using SPSS software (Statistical Package for the Social Sciences, version 25.0; SPSS Inc., Chicago, IL, USA) and R statistical software (version 4.5.2, R Foundation for Statistical Computing, Vienna, Austria). Continuous variables were assessed for normality using the Kolmogorov–Smirnov and Shapiro–Wilk tests. Normally distributed variables were presented as mean ± standard deviation, while non-normally distributed variables were reported as median (minimum–maximum). Categorical variables were summarized as counts and percentages. All statistical tests were two-sided, and a *p* value <0.05 was considered statistically significant.

Survival curves were estimated using the Kaplan–Meier method and compared between groups using the log-rank test. Median OS and 12-month OS rates were reported with corresponding 95% confidence intervals.

For patient-based survival analyses, only the first targeted therapy received by each patient was considered. Event-based analyses were additionally performed to evaluate treatment-specific outcomes. In event-based analyses, each treatment course was analyzed as a separate observation. Events without response assessment due to early discontinuation or insufficient follow-up were excluded from response analyses. Results should therefore be interpreted in the context of available data. Safety analyses were conducted on a per–treatment-event basis. Cytogenetic variables, including del (17p) status, were defined at the patient level and carried forward across treatment events for survival analyses. The association between baseline prognostic factors and overall survival was explored using univariate Cox proportional hazards regression models. Hazard ratios (HRs) with 95% confidence intervals (CIs) and *p* values were reported. Survival analyses were conducted using the survival and survminer packages in R. This study was not designed as a comparative effectiveness analysis. Given the retrospective design, non-randomized treatment allocation, and baseline imbalances across groups, between-group findings were presented descriptively and should not be interpreted as evidence of comparative superiority or differential safety. Because this was a retrospective study and response assessments were not uniformly available in routine clinical practice, response analyses were restricted to treatment events with evaluable response data and do not represent an intention-to-treat analysis.

## 3. Results

### 3.1. Characteristics and Laboratory Results of the Patients

A total of 58 targeted therapy courses were administered to 47 patients with chronic lymphocytic leukemia. Among the 47 patients included in the study, 19 (40.4%) received targeted therapy in the frontline setting, whereas 28 (59.6%) were treated in the relapsed or refractory setting. On a treatment-event basis, obinutuzumab-based therapy accounted for 13 frontline events and 7 relapsed/refractory events, whereas ibrutinib accounted for 5 frontline events and 22 relapsed/refractory events, and venetoclax accounted for 2 frontline events and 9 relapsed/refractory events.

Obinutuzumab, ibrutinib, and venetoclax accounted for 20 (34.5%), 27 (46.5%), and 11 (19.0%) treatment events, respectively. Nine patients received two different targeted therapies, and one patient was treated sequentially with all three agents. Treatment events are summarized in Table 1.

Twenty-eight patients were male (59.5%) and nineteen were female (40.5%), with a male-to-female ratio of 1.47. Sex distribution did not differ significantly between treatment groups. The median age at treatment initiation was 70 years (range, 41–84). Patients receiving obinutuzumab were significantly older compared with those treated with ibrutinib or venetoclax (median age 74 vs. 60 and 61 years, respectively; *p* = 0.001) (Table 2).

The median ECOG performance status was 0 (range, 0–2) and was comparable across treatment groups (*p* > 0.05). Hypertension was the most common comorbidity (63.8%), followed by pulmonary disease (47.7%), coronary artery disease (25.5%), diabetes mellitus (23.4%), prior malignancy (17.0%), and cardiac arrhythmia (10.6%). Hypertension was more frequent among patients treated with obinutuzumab compared with other therapies (75% vs. 44% in the ibrutinib group and 27.3% in the venetoclax group; *p* = 0.02).

**Figure 1:**
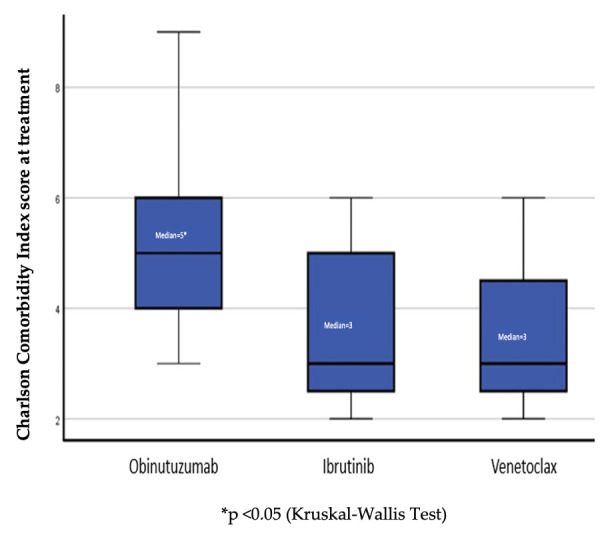
Charlson Comorbidity Index scores according to treatment group. Boxplots show the median, interquartile range, and range of Charlson Comorbidity Index scores in the obinutuzumab, ibrutinib, and venetoclax groups. Between-group differences were evaluated using the Kruskal–Wallis test (*p* = 0.03).

The median CIRS score was 6 (range, 1–18) and was significantly higher in the obinutuzumab group than in the ibrutinib and venetoclax groups (*p* = 0.02). In parallel, the Charlson Comorbidity Index was also significantly higher in patients treated with obinutuzumab (*p* = 0.03) (Figure 1, Table 2).

Advanced-stage disease (Rai stage III–IV or Binet stage C) was the most frequent indication for treatment initiation, accounting for 68.9% of treatment events. Other indications included progressive lymphadenopathy or organomegaly (46.5%) and a lymphocyte doubling time (LDT) of less than six months (22.4%). Treatment indications and disease stage were similar across treatment groups (Table 2).

Baseline clinical and laboratory characteristics were primarily evaluated on a treatment-event basis, whereas survival analyses were conducted on a patient-based dataset considering only the first targeted therapy. In patient-based univariate Cox regression analyses, none of the evaluated baseline clinical or biological variables were significantly associated with overall survival. However, these findings should not be interpreted as evidence of absent prognostic value, since the limited sample size and low number of events likely rendered the analysis underpowered to detect modest associations. Age at treatment initiation, ECOG performance status, comorbidity burden assessed by CIRS, baseline LDH levels, Binet stage, and del (17p) status were not significantly associated with overall survival (Supplementary Table S1).

The median pre-treatment lymphocyte count and hemoglobin level were 67,100/µL (range, 7600–164,200) and 9.5 g/dL (range, 8.6–11.1), respectively. Platelet counts were normally distributed, with a mean value of 124,000 ± 70,000/µL. Hemoglobin levels below 10 g/dL were observed in 34 treatment events (58.6%), and platelet counts below 100,000/µL were recorded in 24 events (41.3%). β2-microglobulin levels were available in 25% of patients and were elevated in all assessed cases; however, missingness precluded formal analysis. Lactate dehydrogenase (LDH) levels were elevated in 21 events (36.2%), with a median value of 216 IU/L (range, 189–322). Although LDH, β2-microglobulin, and lymphocyte levels showed a numerical increase with advancing Rai stage, this association did not reach statistical significance (*p* = 0.054). Laboratory findings are summarized in Table 3. The mean estimated glomerular filtration rate (eGFR), calculated using the Modification of Diet in Renal Disease (MDRD) equation, was 77 ± 35 mL/min. eGFR values were numerically lower in patients treated with obinutuzumab; however, this difference did not reach statistical significance. Renal function was further assessed using Cockcroft–Gault-estimated creatinine clearance, as detailed in Table 3.

Cytogenetic testing for del (17p) was available for 96.5% of treatment events, and del (17p) was detected in 8 events (13.8%). Data on other cytogenetic and molecular abnormalities, including del (11q), trisomy 12, del (13q), and IGHV mutational status, were available for only a limited number of patients and were therefore not included in the statistical analyses.

#### 3.1.1. Efficacy

**Figure 2:**
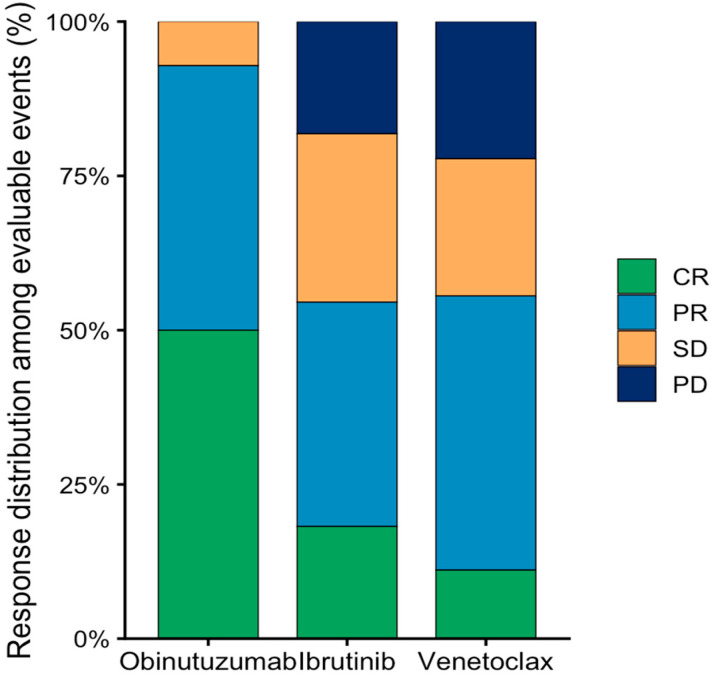
Distribution of treatment responses among response-evaluable targeted therapy events according to targeted agents.

Response assessment was unavailable in 13 events, including 6 obinutuzumab-treated events in the relapsed/refractory setting, 5 ibrutinib-treated events in the frontline setting, and 2 venetoclax-treated events in the frontline setting. These events were considered non-evaluable because treatment had been newly initiated, was still ongoing at the time of data cutoff, follow-up was insufficient for formal response assessment, or no formal response evaluation had yet been documented in routine clinical practice (Supplementary Table S3). Accordingly, the reported response outcomes are based on the 45 response-evaluable treatment events and should be interpreted cautiously. Among response-evaluable treatment events (*n* = 45), the overall response rate was 66.7%. On a per-treatment-event basis, numerically higher response rates were observed in obinutuzumab-treated events (92.9%) than in ibrutinib- (54.5%) and venetoclax-treated events (55.6%) (Table 4, Figure 2). However, these findings should be interpreted descriptively because treatment allocation was not randomized and treatment line was not balanced across groups. In the response-evaluable cohort, all but one obinutuzumab-evaluable event occurred in the frontline setting, whereas evaluable ibrutinib- and venetoclax-treated events were mainly from the relapsed/refractory setting. The observed differences in response are therefore more likely to reflect treatment setting and physician treatment selection than intrinsic differences between agents.

In the overall response-evaluable cohort, complete response, partial response, stable disease, and progressive disease rates were 26.7%, 40.0%, 20.0%, and 13.3%, respectively. Complete responses were more frequent among treatment events involving obinutuzumab, whereas partial responses were observed predominantly with ibrutinib and venetoclax. No disease progression was observed among treatment events achieving at least a partial response at the time of response assessment.

In exploratory subgroup analyses, no clear descriptive pattern in overall response rate was observed according to time from diagnosis to initiation of targeted therapy, treatment line, sex, baseline hematologic or biochemical parameters, Charlson Comorbidity Index score, clinical stage, renal function parameters, or the presence of del (17p); however, these findings should be interpreted cautiously given the limited sample size.

**Figure 3:**
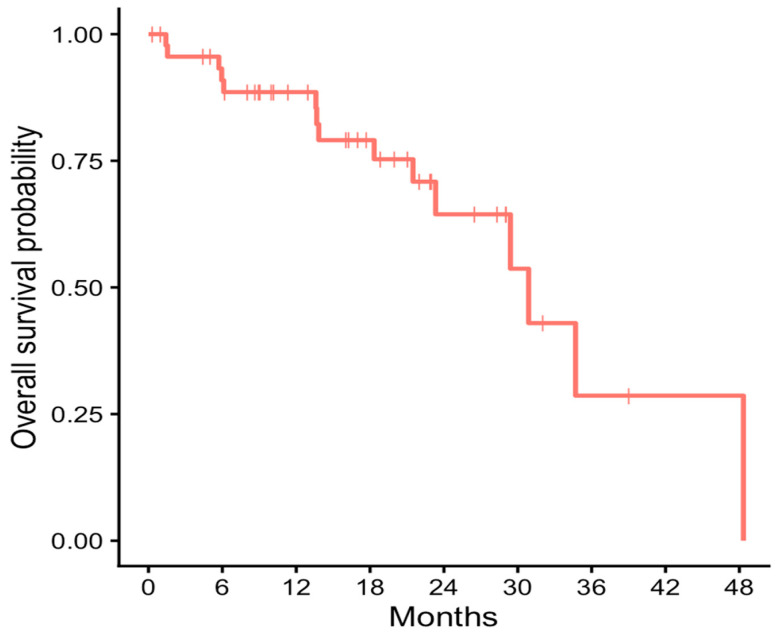
Kaplan–Meier estimate of overall survival in the overall patient cohort.

Overall survival was evaluated at the patient level in the entire cohort (*n* = 47). With a median follow-up of 16.3 months, the median overall survival was 30.9 months (95% confidence interval, 23.3 months to not reached). The estimated 12-month overall survival rate was 88.6% (95% confidence interval, 79.6–98.5), as calculated using the Kaplan–Meier method (Figure 3).

**Figure 4:**
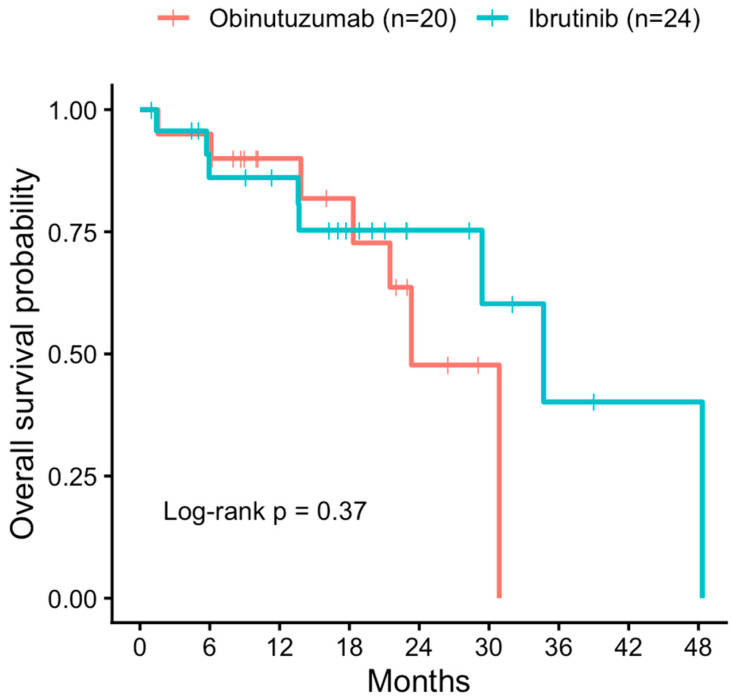
Kaplan–Meier curves showing overall survival from initiation of targeted therapy in patients receiving obinutuzumab or ibrutinib.

In an exploratory analysis limited to patients treated with obinutuzumab or ibrutinib, Kaplan–Meier estimates of overall survival are shown in Figure 4. Overall survival appeared broadly similar between the two treatment groups, although these findings should be interpreted cautiously given the limited sample size and the non-randomized nature of treatment selection.

When examined by treatment type, median overall survival was 23.3 months in patients treated with obinutuzumab and 34.7 months in those receiving ibrutinib. Because the venetoclax group included only 11 treatment events and had limited follow-up, treatment-specific survival estimates for this subgroup were not considered sufficiently robust for detailed interpretation. Accordingly, these results are presented only for descriptive completeness in the supplementary material (Supplementary Figure S1 and Supplementary Table S2) and should be interpreted with particular caution.

No clear descriptive difference in overall survival was observed according to treatment type, duration from diagnosis to targeted therapy initiation, treatment line (first-line or later), gender, baseline biochemical and hematologic parameters, CCI score, clinical stage, creatinine clearance, or the presence of 17p deletion; however, these analyses should be interpreted cautiously given the limited sample size and number of events.

Only response-evaluable treatment events were included in ORR calculations. Events with ongoing treatment or treatment discontinuation due to adverse events were excluded. Findings are presented descriptively because treatment allocation was non-randomized and treatment line was not balanced across groups.

#### 3.1.2. Safety

Adverse events were commonly observed across all treatment groups and are summarized in Table 5. Overall, infections, hematologic toxicities, and bleeding events represented the most frequently reported adverse events. Unless otherwise stated, safety analyses were conducted on a treatment-event basis.

Infectious adverse events were observed in 55.1% of all treatment events, including low-grade (grade 1–2) infections in 29.3% and high-grade (grade 3–4) infections in 25.8%, with broadly similar overall frequencies across treatment groups. Pneumonia and urinary tract infections were the most frequently reported infectious complications across all therapies. High-grade infections were more commonly observed in treatment events involving obinutuzumab and venetoclax.

Neutropenia was among the most frequent hematologic adverse events, occurring in 62.0% of all treatment events, with grade 3–4 neutropenia reported in 48.2%**.** High-grade neutropenia appeared more frequent in treatment events involving obinutuzumab (60.0%) and venetoclax (72.7%); however, given the non-randomized treatment allocation and baseline imbalances across groups, these findings should be interpreted descriptively. Febrile neutropenia occurred in a subset of patients, most frequently among those treated with venetoclax.

Thrombocytopenia was observed in 51.6% of treatment events, with broadly similar descriptive distributions of low-grade and high-grade events across treatment groups. Grade 3–4 thrombocytopenia occurred in 30.0% of obinutuzumab-treated events, 14.8% of ibrutinib-treated events, and 45.4% of venetoclax-treated events.

Bleeding was observed in 11 of 27 ibrutinib-treated events (40.7%), whereas no bleeding events were observed with obinutuzumab or venetoclax. Of these, seven were low-grade events (grade 1–2) and three were grade 3–4. In addition, one patient died due to grade 5 intracerebral hemorrhage. Minor bleeding was mainly limited to mucocutaneous events, whereas severe cases included hematuria and subscapular hematoma.

Infusion-related reactions were observed in 11 of 20 obinutuzumab-treated events (55.0%), including grade 1–2 reactions in 6 events (30.0%) and grade 3–4 reactions in 5 events (25.0%).

Other adverse events, including tumor lysis syndrome, hepatotoxicity, and dermatologic reactions, were infrequently observed across treatment groups. No unexpected safety signals were identified during the study period. Given the limited number of treatment events, particularly in the venetoclax group, these findings should be interpreted with caution. Overall, the safety profiles observed were consistent with the known toxicity patterns of the respective agents in real-world clinical practice.

Treatment discontinuation due to infusion-related anaphylaxis occurred in three patients receiving obinutuzumab, corresponding to 15% of patients in this group. Among patients treated with ibrutinib, treatment was discontinued in four patients (14.8%) due to bleeding complications and in one patient (3.7%) due to newly developed atrial fibrillation. In the venetoclax-treated group, treatment discontinuation was observed in one patient (9%) as a result of recurrent neutropenia accompanied by febrile neutropenia.

Findings are presented descriptively because treatment allocation was non-randomized and baseline characteristics differed across groups. Accordingly, formal inferential interpretation of between-group efficacy and safety differences was avoided.

## 4. Discussion

The introduction of targeted therapies has substantially changed the management of chronic lymphocytic leukemia; however, data regarding their efficacy and safety in routine clinical practice remain limited. This is particularly relevant for real-world populations, which often include older patients with significant comorbidities and heterogeneous treatment histories. In this retrospective study, we evaluated the clinical outcomes of patients with CLL treated with obinutuzumab, ibrutinib, or venetoclax outside the setting of prospective clinical trials and within routine clinical practice.

The demographic characteristics of our cohort were consistent with previously published epidemiological data, with a predominance of male patients and a median age at treatment initiation of 70 years [1,12]. Patients treated with obinutuzumab were significantly older and had a higher comorbidity burden compared with those receiving ibrutinib or venetoclax. Similar findings have been reported in both randomized trials and real-world studies, where obinutuzumab-based regimens were preferentially administered to elderly patients or those with multiple comorbidities [5,13,14,15]. These observations suggest that obinutuzumab is frequently selected in clinical practice for patients considered less suitable for continuous kinase inhibitor therapy. Similar treatment patterns have been described in published real-world series, in which obinutuzumab-based regimens were used more often in older and more comorbid patients, while ibrutinib- and venetoclax-based approaches were more frequently given in different clinical settings, particularly in relapsed/refractory disease [13,14,15,16,17]. This supports the view that our cohort reflects routine treatment selection in daily practice rather than a trial-selected population.

In our cohort, numerically higher response rates were observed in treatment events involving obinutuzumab compared with ibrutinib and venetoclax. This should be interpreted with caution, since obinutuzumab was used more often in the first-line setting, whereas ibrutinib and venetoclax were administered mainly in relapsed/refractory disease. In the response-evaluable subgroup, all but one obinutuzumab-evaluable event occurred in the frontline setting. This pattern suggests that the observed response differences were influenced largely by treatment setting and patient selection, rather than by the drugs themselves. High response rates with obinutuzumab have been consistently reported in treatment-naïve populations, including a real-world series by Gay et al., in which all patients were previously untreated and achieved high overall and complete response rates [18]. Survival outcomes observed in our obinutuzumab-treated patients were within the range reported in previous real-world and clinical studies [5,15].

Response and survival outcomes in patients treated with ibrutinib and venetoclax were numerically lower in our cohort. These findings likely reflect the predominance of heavily pretreated and relapsed/refractory patients in these groups, a population known to exhibit inferior response and survival outcomes. Indeed, lower response rates and shorter survival have been consistently reported in real-world cohorts enriched for patients with advanced disease and multiple prior lines of therapy, in comparison with pivotal clinical trials conducted in more selected populations [6,19,20,21]. In routine clinical practice, observed differences in treatment effectiveness across agents in our cohort appear to be primarily driven by patient selection and prior treatment exposure rather than intrinsic differences in drug efficacy.

Although median OS estimates varied across treatment groups, these differences should be interpreted in the context of baseline imbalances and prior treatment exposure. Early survival rates were broadly comparable and remained within the range reported in selected real-world series [14,16]. The relatively shorter median OS observed in the ibrutinib and venetoclax groups is likely attributable to the predominance of heavily pretreated and relapsed/refractory patients, a population known to have inferior long-term outcomes compared with clinical trial populations [19,20,22]. Twelve-month OS rates were relatively high across treatment groups in this cohort [13,14].

Hypertension was the most common comorbidity in our cohort and was significantly more prevalent among patients treated with obinutuzumab. Comparable rates have been reported in previous studies evaluating obinutuzumab-based therapies in elderly or comorbid populations [5,13]. In routine practice, this preference may also reflect the tendency to favor a supervised intravenous regimen in selected frail older patients, particularly when long-term adherence or tolerability of continuous oral treatment is a concern. In contrast, ibrutinib was used less frequently in patients with cardiovascular comorbidities, which likely reflects clinical concerns regarding bleeding risk and cardiac toxicity [16,19]. This treatment pattern is consistent with real-world prescribing behavior and reflects the influence of comorbidity burden on treatment selection.

In line with this prescribing pattern, bleeding events emerged as a prominent agent-specific adverse effect among patients treated with ibrutinib in our cohort. Bleeding complications were observed more often in ibrutinib-treated events than in obinutuzumab- or venetoclax-treated events in our cohort, although these findings are descriptive given the non-randomized treatment allocation. The spectrum of bleeding events ranged from low-grade mucocutaneous bleeding to severe hemorrhagic complications, including intracranial bleeding, leading to treatment discontinuation in a subset of patients. These findings are consistent with previously published clinical trial and real-world data demonstrating an increased risk of bleeding with Bruton tyrosine kinase inhibition, likely related to platelet dysfunction and impaired collagen-mediated platelet aggregation [16,19,23,24]. In routine clinical practice, this risk often influences treatment selection, particularly in patients with underlying cardiovascular disease or those requiring antiplatelet or anticoagulant therapy. Our findings reinforce the importance of careful patient selection and vigilant monitoring when ibrutinib is prescribed to patients with an increased baseline risk of bleeding.

In the obinutuzumab-treated group, infusion-related reactions represented the most characteristic adverse events, with severe reactions leading to treatment discontinuation in a small subset of patients. These findings are consistent with the established safety profile of obinutuzumab reported in randomized clinical trials and international treatment guidelines and are particularly relevant in elderly and comorbid patients, who comprised a substantial proportion of our cohort [5,25]. These observations suggest that obinutuzumab was frequently selected for older and more comorbid patients in routine practice, reflecting physician treatment preferences [25].

In patients treated with venetoclax, hematologic toxicities—particularly neutropenia and febrile neutropenia—were the most frequently observed adverse events. Although high-grade neutropenia occurred in a substantial proportion of patients, most events were manageable with close monitoring and supportive care, including secondary G-CSF prophylaxis, and rarely necessitated permanent treatment discontinuation. These observations are consistent with previously reported clinical trial and guideline data, which emphasize the predictable and manageable nature of venetoclax-associated hematologic toxicity when appropriate preventive and supportive strategies are implemented [17,20,25].

Overall, despite the frequent occurrence of adverse events, treatment discontinuation was uncommon and was largely attributable to agent-specific toxicities rather than generalized treatment intolerance, reflecting the feasibility of these therapies in routine clinical practice [5,19,20].

This study has limitations inherent in its retrospective design, including potential selection bias and incomplete documentation of adverse events. In addition, response assessment was not available for 13 treatment events because treatment had just been started, was still ongoing at the time of data cutoff, or follow-up was not sufficient for formal evaluation. This may have affected the reported response rates and should be taken into account when interpreting the efficacy findings. The small sample size and treatment heterogeneity, particularly with respect to line of therapy, limit the generalizability of these findings and restrict direct cross-agent comparisons. In particular, survival estimates for the venetoclax subgroup were not sufficiently robust for detailed interpretation because of the small number of treatment events and limited follow-up. In addition, outcomes were assessed in routine practice rather than under protocolized trial conditions. Nevertheless, the cohort reflects an older, comorbid population frequently underrepresented in clinical trials, offering descriptive insights into treatment selection and tolerability in routine clinical practice.

## 5. Conclusions

In this real-world cohort of patients with chronic lymphocytic leukemia, targeted therapies showed response patterns and safety findings consistent with routine clinical practice. Observed differences across treatment groups likely reflected patient selection and prior treatment exposure rather than direct comparative efficacy. These findings provide descriptive insight into real-world prescribing patterns and may serve as hypothesis-generating observations for future prospective studies.

## Figures and Tables

**Table 1:** Treatment events.

Treatment Drug	Total *n* (%)	First Line, *n*	R/R, *n*
Obinutuzumab	20 (34.5)	13	7
Monotherapy	16	9	7
Combination with chlorambucil	4	4	0
Venetoclax	11 (19.0)	2	9
Monotherapy	7	2	5
Combination with rituximab	4	0	4
Ibrutinib	27 (46.5)	5	22

First-line was defined as the first systemic treatment administered for CLL. R/R was defined as treatment given for relapsed or refractory disease after at least one prior line of therapy.

**Table 2:** Pre-treatment demographic and clinical characteristics of patients.

Parameters	Total Events (*n* = 58)	Obinutuzumab Events (*n* = 20)	Ibrutinib Events (*n* = 27)	Venetoclax Events (*n* = 11)	*p* Value
**Age, median (IQR)**	70 (55–76)	74 (71–79)	61 (53–73)	60 (51–71)	**0.001 ^a^**
**Hepatomegaly, *n* (%)**	44 (75.9)	13 (65)	21 (77.8)	10 (90.9)	0.2 ^b^
**Splenomegaly, *n* (%)**	50 (86.2)	17 (85)	24 (88.9)	9 (81.8)	0.8 ^c^
**Liver size (mm), mean ± SD**	173 ± 25	169 ± 29	172 ± 26	177 ± 22	0.7 ^d^
**Spleen size (mm), mean ± SD**	168 ± 36	168 ± 40	169 ± 32	153 ± 36	0.4 ^d^
**B symptom**					
**Yes (*n*, %)** **No (*n*, %)**	34 (58.6) 24 (41.4)	13 (65) 7 (35)	16 (59.2) 11 (40.8)	5 (45.5) 6 (54.5)	0.4 ^b^
**ECOG score**					
**ECOG 0** **ECOG 1** **ECOG 2**	38 (65.5) 14 (24.1) 6 (10.3)	13 (65) 5 (25) 2 (10)	19 (70.4) 5 (18.5) 3 (11.1)	6 (54.5) 4 (36.4) 1 (9.1)	0.8 ^c^
**RAI Stage**					
**Low risk (Stage 0)** **Intermediate risk (Stage 1–2)** **High risk (Stage 3–4)**	0 10 (17.2) 48 (82.8)	0 2 (10) 18 (90)	0 6 (22.2) 21 (77.8)	0 2 (18.2) 9 (81.8)	0.5 ^c^
**BINET Stage**					
**Stage A** **Stage B** **Stage C**	0 14 (24.1) 44 (75.9)	0 4 (20) 16 (80)	0 8 (29.6) 19 (70.4)	0 2 (18.2) 9 (81.8)	0.6 ^b^
**Charlson Comorbidity** **Index Score, median**	3 (2–4)	5 (3–9)	3 (2–6)	3 (2–6)	**0.03 ^a^**
**CIRS, median (IQR)**	5 (3–9)	7 (5–11)	5 (3–9)	4 (3–7)	**0.02 ^a^**

SD: Standard Deviation, IQR: interquantile range ^a^: Kruskal–Wallis. ^b^: Chi-square. ^c^: Fisher’s exact test. ^d^: One-way ANOVA.

**Table 3:** The laboratory parameters of the patients before every event.

Parameters	Total Events (*n* = 58)	Obinutuzumab (*n* = 20)	Ibrutinib (*n* = 27)	Venetoclax (*n* = 11)	*p* Value
**Hemoglobin (g/dL), median (IQR)**	9.5 (8.6–11.1)	9.2 (8.7–10.4)	9 (8.4–11.8)	10 (8.6–11.7)	0.5 ^b^
**Leukocyte (×10^3^/µL), median (IQR)**	73.8 (10.9–171)	89.8 (13.5–197.5)	76.1 (16.8–187.5)	28.3 (3.8–134.4)	0.2 ^b^
**Lymphocyte (×10^3^/µL), median (IQR)**	67.1 (7.6–164.2)	81.1 (5–184.3)	70.2 (14.1–181.4)	27.2 (2.3–130)	0.3 ^b^
**Neutrophil (×10^3^/µL), median (IQR)**	4.7 (2.1–17.3)	5.6 (2.8–9.9)	4.7 (2.1–7.9)	2.7 (0.8–4.3)	0.1 ^b^
**Platelets (×10^3^/µL), mean ± SD**	124 ± 70	132 ± 59	123 ± 80	115 ± 64	0.8 ^a^
**LDH (IU/L), median (IQR)**	216 (189–322)	225 (183–308)	223 (186–346)	214 (195–305)	0.9 ^b^
**Albumin (g/dL)**	3.8 ± 0.5	3.7 ± 0.6	4 ± 0.4	3.6 ± 0.3	0.054 ^a^
**eGFR (MDRD), mean ± SD (mL/min)** **CrCl (Cockcroft–Gault), mean ± SD (mL/min)**	83 ± 27 83 ± 26	77 ± 35 72 ± 27	86 ± 22 88 ± 25	91 ± 19 98 ± 13	0.5 ^a^ 0.1 ^a^

SD: Standard deviation, IQR: interquantile range, eGFR: estimated glomerular filtration rate, CrCl: Creatinine clearance, MDRD: Modification Diet in Renal Disease, ^a^: One-way ANOVA ^b^: Kruskal–Wallis.

**Table 4:** Treatment response according to targeted therapy (response-evaluable treatment events).

Treatment	Evaluable Events (*n*)	CR	PR	SD	PD	ORR (%)
Obinutuzumab	14	7	6	1	0	92.9
Ibrutinib	22	4	8	6	4	54.5
Venetoclax	9	1	4	2	2	55.6

**Table 5:** Adverse events profile in Obinutuzumab, Ibrutinib, and Venetoclax.

Adverse Events	Total Events (*n* = 58)	Obinutuzumab (*n* = 20)	Ibrutinib (*n* = 27)	Venetoclax (*n* = 11)
**Infections, *n* (%)**				
**Low grade (grade 1 and 2)** **High grade (grade 3 and 4)**	17 (29.3) 15 (25.8)	5 (25) 6 (30)	8 (29.6) 6 (22.2)	4 (36.3) 3 (27.2)
**Neutropenia, *n* (%)**				
**Low grade (grade 1 and 2)** **High grade (grade 3 and 4)**	8 (13.7) 28 (48.2)	4 (20) 12 (60)	3 (11.1) 8 (29.6)	1 (9) 8 (72.7)
**Thrombocytopenia, *n* (%)**				
**Low grade (grade 1 and 2)** **High grade (grade 3 and 4)**	15 (25.8) 15 (25.8)	6 (30) 6 (30)	5 (18.5) 4 (14.8)	4 (36.3) 5 (45.4)
**Others, *n* (%)**				
**Infusion-related reaction, *n* (%)** **Bleeding, *n* (%)** **Tumor lysis syndrome, *n* (%)** **Hepatotoxicity, *n* (%)** **Dermatologic, *n* (%)**	11 (18.9) 11 (18.9) 7 (12) 1 (1.7) 1 (1.7)	11 (55) 0 3 (15) 1 (5) 0	0 11 (40.7) 2 (7.4) 0 0	0 0 2 (18.1) 0 1 (9)

## Data Availability

The data supporting the findings of this study are not publicly available due to patient privacy and ethical restrictions but are available from the corresponding author upon reasonable request.

## Supplementary Materials

The following supporting information can be downloaded at: https://www.mdpi.com/article/10.3390/medicina62040736/s1. Figure S1: Kaplan–Meier curves of overall survival stratified by treatment group. Table S1: Univariate Cox regression analysis of baseline prognostic factors associated with overall survival (patient-level analysis). Table S2: Twelve-month overall survival estimates according to targeted therapy. Supplementary Table S3: Distribution and reasons for exclusion of non-evaluable treatment events from the response analysis.

## Abbreviations

The following abbreviations are used in this manuscript:

CLL	Chronic Lymphocytic Leukemia
OS	Overall Survival
BTK	Bruton Tyrosine Kinase
G-CSF	Granulocyte Colony-Stimulating Factor
ECOG	Eastern Cooperative Oncology Group
CIRS	Cumulative Illness Rating Scale
