# Supplementary material for: Real-World Treatment Patterns and Safety Outcomes of Targeted Therapies in a Single-Center Chronic Lymphocytic Leukemia Cohort
Source: Medicina (Kaunas). 2026 Apr 12;62(4):736. doi: 10.3390/medicina62040736 (PMC13117187; doi:10.3390/medicina62040736)
Supplement: Supplementary file 1 [file medicina-62-00736-s001.zip › supplement materials.pdf]

Supplementary Figure S1.
Kaplan–Meier curves of overall survival stratified by treatment type. Overall survival was measured from the initiation of first targeted therapy. Shaded areas indicate 95% confidence intervals.

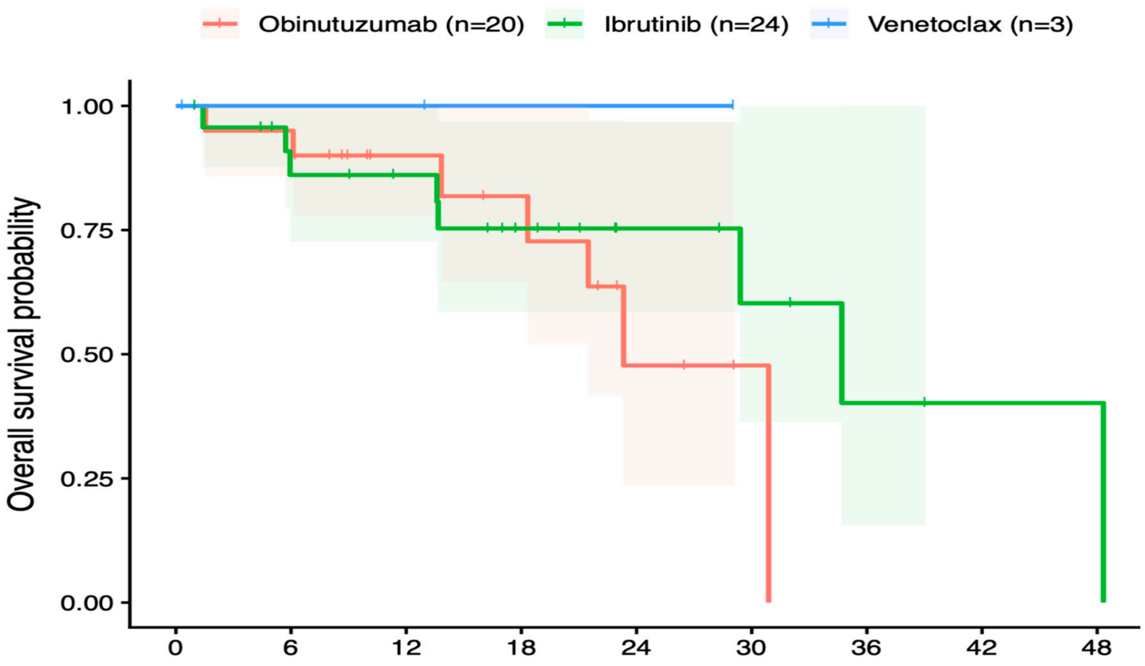

Supplementary Table S1.
Univariate Cox regression analysis of baseline prognostic factors for overall survival (patient-based analysis).

Variable	HR	95% CI	p-value
Age at targeted therapy (≥70 vs <70)	1.05	0.36–3.06	0.92
ECOG ≥2 vs 0–1	1.35	0.29–6.36	0.70
CIRS ≥6 vs <6	0.79	0.27–2.31	0.67
Elevated LDH (yes vs no)	1.70	0.60–4.88	0.32
Binet stage (B–C vs A)	0.79	0.27–2.31	0.67
del(17p) (present vs absent)	0.72	0.09–5.72	0.76

Supplementary Table S2. Twelve-month overall survival according to targeted therapy

Treatment	12-month OS (%)	95% Confidence Interval	Patients at risk at 12 months
Obinutuzumab	90.0	77.8–100.0	11
Ibrutinib	86.1	72.6–100.0	16
Venetoclax	100.0	100.0–100.0	2
