# Supplementary material for: Real-World Treatment Patterns and Safety Outcomes of Targeted Therapies in a Single-Center Chronic Lymphocytic Leukemia Cohort
Source: Medicina (Kaunas). 2026 Apr 12;62(4):736. doi: 10.3390/medicina62040736 (PMC13117187; doi:10.3390/medicina62040736)
Supplement: Supplementary file 1 [file medicina-62-00736-s001.zip › Supplementary Tables.pdf]

Supplementary Table S1.
Univariate Cox regression analysis of baseline prognostic factors for overall survival (patient-based analysis).

Variable	HR	95% CI	p-value
Age at targeted therapy (≥70 vs <70)	1.05	0.36–3.06	0.92
ECOG ≥2 vs 0–1	1.35	0.29–6.36	0.70
CIRS ≥6 vs <6	0.79	0.27–2.31	0.67
Elevated LDH (yes vs no)	1.70	0.60–4.88	0.32
Binet stage (B–C vs A)	0.79	0.27–2.31	0.67
Del (17p) (present vs absent)	0.72	0.09–5.72	0.76

Supplementary Table S2. Twelve-month overall survival according to targeted therapy

Treatment	12-month OS (%)	95% Confidence Interval	Patients at risk at 12 months
Obinutuzumab	90.0	77.8–100.0	11
Ibrutinib	86.1	72.6–100.0	16
Venetoclax	100.0	100.0–100.0	2

Supplementary Table S3. Distribution and reasons for exclusion of non-evaluable treatment events from the response analysis

Treatment agent	Treatment setting	Number of non-evaluable events	Reason for exclusion
Obinutuzumab	Relapsed/refractory	2	Treatment had been newly initiated and formal response assessment was not yet available.
Obinutuzumab	Relapsed/refractory	3	Treatment was ongoing at the time of data cutoff without a documented formal response evaluation.
Obinutuzumab	Relapsed/refractory	1	Follow-up duration was insufficient for response assessment according to routine clinical practice.
Ibrutinib	Frontline	2	Treatment was ongoing at the time of data cutoff and no formal response assessment had yet been documented.
Ibrutinib	Frontline	2	Follow-up was too short for a reliable response evaluation.
Ibrutinib	Frontline	1	Response had not yet been formally assessed in the medical record despite ongoing therapy.
Venetoclax	Frontline	1	Treatment was in the early phase and formal response assessment was not yet available.
Venetoclax	Frontline	1	Treatment was ongoing at data cutoff before scheduled response evaluation.
