# Supplementary figures and images for: Real-World Treatment Patterns and Safety Outcomes of Targeted Therapies in a Single-Center Chronic Lymphocytic Leukemia Cohort
Source: Medicina (Kaunas). 2026 Apr 12;62(4):736. doi: 10.3390/medicina62040736 (PMC13117187; doi:10.3390/medicina62040736)

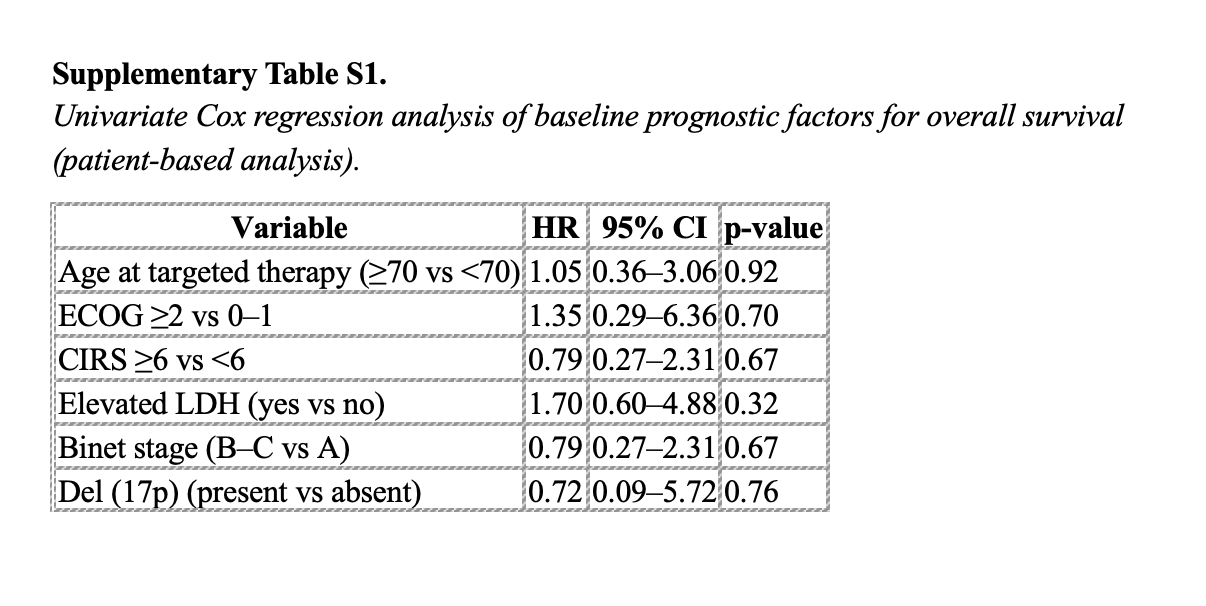

Supplement: Supplementary file 1 [file medicina-62-00736-s001.zip › Table S1.png]

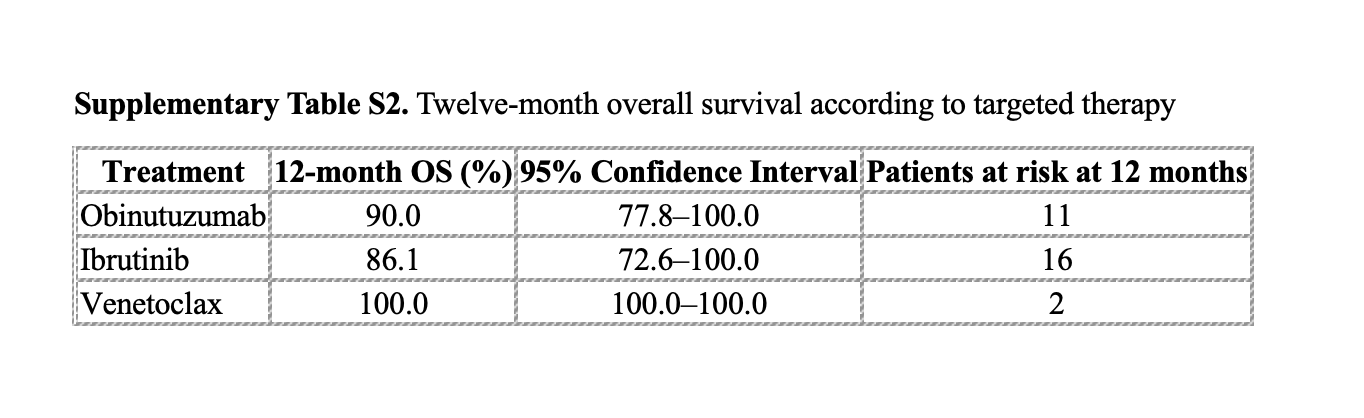

Supplement: Supplementary file 1 [file medicina-62-00736-s001.zip › Table S2.png]

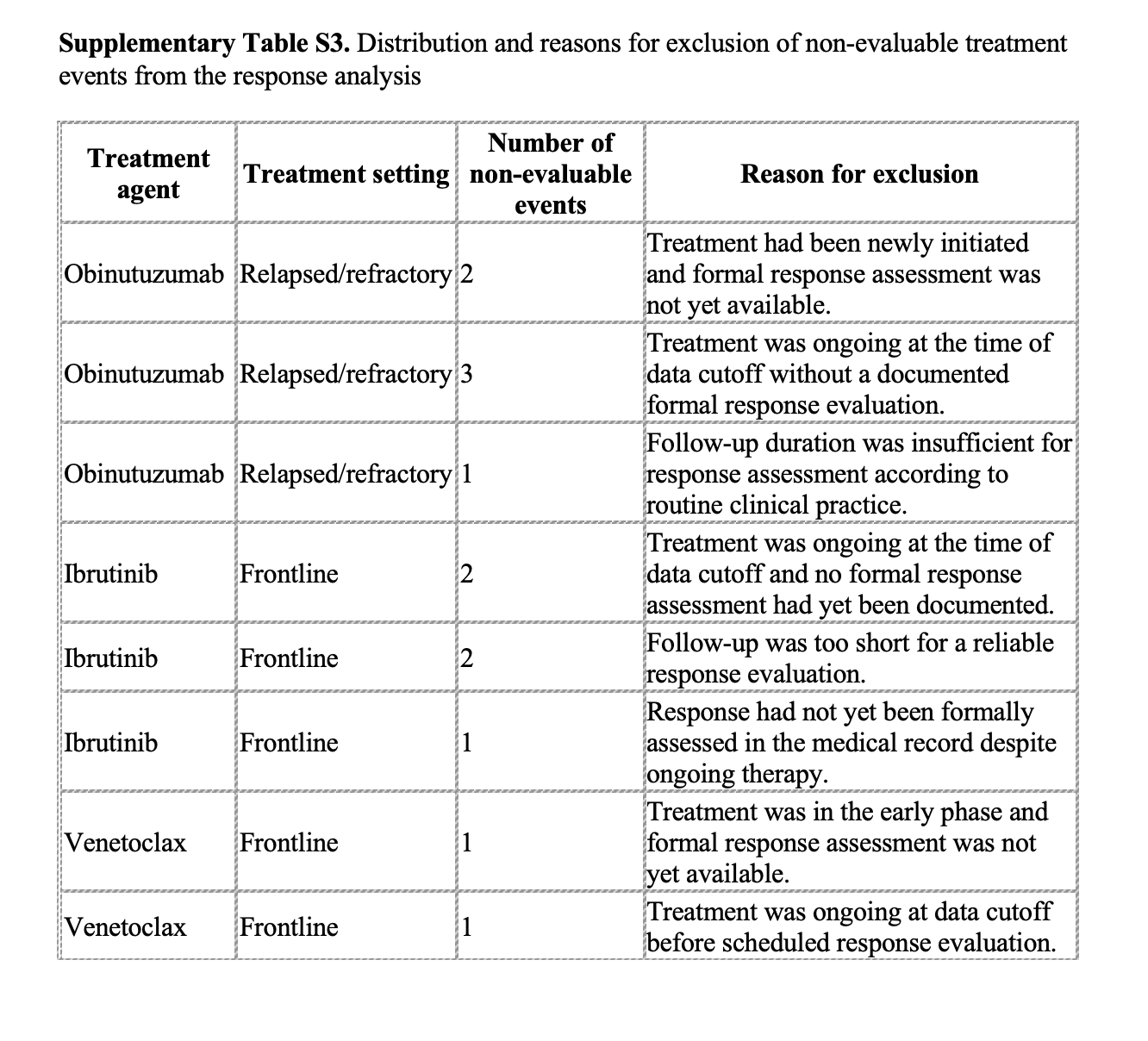

Supplement: Supplementary file 1 [file medicina-62-00736-s001.zip › Table S3.png]
